# HOW DO OLDER ADULTS PRIORITIZE DIFFERENT ASPECTS OF THEIR PHYSICAL HEALTH?

**DOI:** 10.1093/geroni/igae098.3274

**Published:** 2024-12-31

**Authors:** Rachel Logue Cook, Susan Brown

**Affiliations:** University of Michigan, Ann Arbor, Michigan, United States; University of Michigan, Ann Arbor, Michigan, United States

## Abstract

Older adults often manage multiple health concerns and must decide which aspects of health to prioritize. While gerontologists and geriatricians are typically concerned about the development of cancer, cognitive and mobility impairments, and mental health disorders (Kotsani et al., 2021), little is known about which aspects of health are important to older adults. We developed an online survey that asked respondents to rank 10 health areas (arm and shoulder function, balance, bladder and bowel function, hand function, memory and cognition, mental health and mood, sleep, speech and communication, vision, walking) from most (#1) to least (#10) important in their daily life. Participants were also asked to explain why these rankings were selected in open-ended questions. Five hundred and ninety-five older adults (mean age 71.5 ± 5.5y) completed the survey. Health areas most often ranked as #1 were memory and cognition (36%), vision (19%), and mental health and mood (18%). Areas ranked as #10 were most commonly bladder and bowel function (22%), arm and shoulder function (21%), and sleep (21%). Open-ended responses indicated that health areas were given higher priority if respondents felt they impacted their ability to maintain independence, participate in valued activities, or they had experienced an impairment. Results from this study indicate that many older adults consider memory, vision, and mental health as important components of healthy aging. Further, these findings underscore the importance of understanding how and why older adults prioritize their health in order for healthcare providers to better serve the aging population.

